# Constitutive Modeling with Single and Dual Internal Variables

**DOI:** 10.3390/e25050721

**Published:** 2023-04-26

**Authors:** Arkadi Berezovski

**Affiliations:** Department of Cybernetics, School of Science, Tallinn University of Technology, Ehitajate tee 5, 19086 Tallinn, Estonia; arkadi.berezovski@taltech.ee

**Keywords:** internal variables, constitutive modeling, heat conduction, elastic waves, viscous fluids

## Abstract

Phenomenological constitutive models with internal variables have been applied for a wide range of material behavior. The developed models can be classified as related to the single internal variable formalism based on the thermodynamic approach by Coleman and Gurtin. The extension of this theory to so-called dual internal variables opens up new avenues for the constitutive modeling of macroscopic material behavior. This paper reveals the distinction between constitutive modeling with single and dual internal variables using examples of heat conduction in rigid solids, linear thermoelasticity, and viscous fluids. A thermodynamically consistent framework for treating internal variables with as little a priori knowledge as possible is presented. This framework is based on the exploitation of the Clausius–Duhem inequality. Since the considered internal variables are “observable but not controllable”, only the Onsagerian procedure with the use of the extra entropy flux is appropriate for the derivation of evolution equations for internal variables. The key distinctions between single and dual internal variables are that the evolution equations are parabolic in the case of a single internal variable and hyperbolic if dual internal variables are employed.

## 1. Introduction

Constitutive modeling provides material-specific relationships between field quantities complementing balance laws. The emphasis is on developing mathematical models that can describe specific physical phenomena. The effectiveness of constitutive models is critical to the predictive capabilities of material computational models. Classical constitutive models, such as the Fourier law for heat flux and the Hooke law relating stress and strain, provide the description of homogeneous materials. Real materials have a microstructure composed of inhomogeneities of varying sizes, distributions, and properties. Even if the length scale of the microstructure is much smaller than the length scale of a body, the effect of the microstructure on overall body response may not be negligible. As a result, the influence of the microstructure should be considered in the macroscopic material description. Various approaches are used to describe the macroscopic behavior influenced by the internal structure of the materials. Homogenization methods result in the construction of effective media, which take into account the effects of the internal structure, e.g., [1,2,3,4,5,6]. More sophisticated generalized continua theories necessitate a large number of additional material parameters [7,8,9,10,11].

For a wide range of material behavior, phenomenological constitutive models with internal variables have been applied [12,13,14,15,16,17,18,19]. Even if multiple internal variables are introduced, the developed models can be classified as related to the single internal variable formalism based on the thermodynamic approach by Coleman and Gurtin [20]. The extension of this theory to so-called dual internal variables [21] opens up new avenues for the constitutive modeling of macroscopic material behavior.

The paper reveals the distinction between constitutive modeling with single and dual internal variables using examples of heat conduction in rigid solids, linear thermoelasticity, and viscous fluids. The primary challenge in implementing internal variable theory is the formulation of evolution equations for internal variables. The derivation of evolution equations is based on the exploitation of the dissipation inequality. Two common methods for exploiting the dissipation inequality are the Coleman–Noll procedure [22] and the Liu procedure [23]. Both procedures assume that the time rates of the state variables can be chosen arbitrarily. This assumption is not applicable for internal variables of state because they are “observable but not controllable” [13,24], which means that in contrast to dynamic degrees of freedom, internal variables cannot be governed by boundary conditions. Only the Onsagerian procedure with the use of thermodynamic forces and flux relationships is appropriate in this case [25].

We begin with the formulation of local balance laws and move on to the classic constitutive relations. Then, for heat conduction in rigid solids, linear thermoelasticity, and viscous fluids, a single internal variable framework is represented sequentially. The same phenomena are considered in the case of dual internal variables.

The main result of the paper is the demonstration of the ability of the dual internal variable approach to generate a hyperbolic evolution equation for internal variables that is applicable to various physical situations. It is shown in the case of heat conduction that a wave-like heat propagation can be described in a thermodynamically consistent manner. The causality issue for second gradient elasticity is avoided in the case of thermoelasticity. The Maxwell fluids model is recovered in the single internal variable case for viscous fluids, and extended equations of fluid motion are obtained using the dual internal variable approach.

## 2. Balance Laws

The behavior of a continuous medium is governed by balance laws. The local balance laws can be represented in the Eulerian (spatial) representation as follows (cf. [26]):

- Conservation of mass:
  (1) $$\rho_{t}+\rho \;\mathrm{div}\;v=0,$$
  where ρ is the density field, v is the velocity field, and subscript *t* denotes the material time derivative;
- Balance of linear momentum:
  (2) $$\rho v_{t}-\mathrm{div}\;\sigma =\rho f,$$
  where σ is the Cauchy stress tensor and f is a body force;
- Balance of angular momentum:
  (3) $$\sigma^{T}=\sigma ,$$
  where upper index *T* denotes transposition;
- Balance of energy:
  (4) $$\rho e_{t}-\sigma :D+\mathrm{div}\;q=\rho h,$$
  where *e* is the internal energy density, D is the strain rate tensor, q is heat flux, and *h* is a heat body source.

The entropy inequality supplements the balance laws

(5) $$\rho \eta_{t}+\mathrm{div}\left(\frac{q}{\theta }\right)-\rho \frac{h}{\theta }\geq 0,$$

where η is the entropy density field and θ is the absolute temperature.

In what follows, we consider the simplest possible situations:

- Small deviation of temperature from the reference value;
- Small-strain approximation;
- Zero body sources;
- Quadratic free energy.

## 3. Classical Constitutive Relations

As a starting point, we recall the classical constitutive models.

### 3.1. Heat Conduction in Rigid Solids

Because the matter density in rigid solids is constant by definition, dealing with internal energy per unit volume E=ρe and entropy per unit volume S=ρη is more convenient. The free energy per unit volume W=E−θS in rigid solids is only temperature-dependent, e.g., [27]:

(6) W=W(θ).

In the absence of body sources, the local balance law for energy in terms of free energy reads

(7) $$W_{t}+\theta S_{t}+S\theta_{t}+\mathrm{div}q=0.$$

The second law of thermodynamics is written as

(8) $$S_{t}+\mathrm{div}\left(\frac{q}{\theta }\right)\geq 0.$$

Multiplying the second law (8) by temperature:

(9) $$\theta S_{t}+\theta \mathrm{div}\left(\frac{q}{\theta }\right)\geq 0,$$

and comparing the balance of energy (7) with Equation (9), we arrive at the inequality

(10) $$-\left(\frac{\partial W}{\partial \theta }+S\right)\theta_{t}-\frac{q}{\theta }\cdot \nabla \theta \geq 0.$$

Here, “·” denotes the scalar product of vectors.

Due to the arbitrariness of the temperature time rate, entropy per unit volume is defined as

(11) $$S=-\frac{\partial W}{\partial \theta },$$

and the dissipation inequality (10) is reduced to

(12) $$-\frac{q}{\theta }\cdot \nabla \theta \geq 0.$$

This inequality is satisfied by the Fourier law:

(13) q=−k∇θ,

with the thermal conductivity *k* considered here as a positive constant.

If the free energy per unit volume is a quadratic function

(14) $$W=-\frac{\rho c_{p}}{2\theta_{0}}{\left(\theta -\theta_{0}\right)}^{2},$$

where $c_{p}$ is the heat capacity, then the heat conduction equation for small deviations from the reference temperature $\theta_{0}$ takes the classical form

(15) $$\rho c_{p}\theta_{t}-k\Delta \theta =0.$$

Classical heat conduction Equation (15) is valid for homogeneous isotropic materials obeying the Fourier law.

### 3.2. Linear Thermoelasticity

In the case of linear thermoelasticity, the free energy per unit volume depends on the strain tensor ε, the temperature, and, possibly, on the temperature gradient:

(16) W=W(ε,θ,∇θ).

Rewriting the balance of energy in terms of free energy:

(17) $$W_{t}+{\left(\theta S\right)}_{t}-\sigma :\varepsilon_{t}+\mathrm{div}\;q=0,$$

and multiplying the second law by temperature once more:

(18) $$\theta S_{t}+\theta \mathrm{div}\left(\frac{q}{\theta }\right)\geq 0,$$

we obtain the Clausius–Duhem inequality (no body sources) [28]:

(19) $$-W_{t}-S\theta_{t}-\frac{q}{\theta }\cdot \nabla \theta +\sigma :\varepsilon_{t}\geq 0.$$

Here, $\varepsilon_{t}=D$ due to kinematic compatibility conditions. The substitution of the time derivative of the free energy

(20) $$\begin{array}{c} W_{t}=\frac{\partial W}{\partial \varepsilon }\varepsilon_{t}+\frac{\partial W}{\partial \theta }\theta_{t}+\frac{\partial W}{\partial \nabla \theta }{\left(\nabla \theta \right)}_{t}, \end{array}$$

into dissipation inequality (19) results in

(21) $$\begin{array}{c} \left(-\frac{\partial W}{\partial \varepsilon }+\sigma \right)\varepsilon_{t}+\left(-S-\frac{\partial W}{\partial \theta }\right)\theta_{t}-\frac{\partial W}{\partial \nabla \theta }{\left(\nabla \theta \right)}_{t}-\frac{q}{\theta }\cdot \nabla \theta \geq 0. \end{array}$$

The arbitrariness of the time rates for strain, temperature, and its gradient yields

(22) $$\begin{array}{c} \left(-\frac{\partial W}{\partial \varepsilon }+\sigma \right)=0,\;\left(-S-\frac{\partial W}{\partial \theta }\right)=0,\;\frac{\partial W}{\partial \nabla \theta }=0. \end{array}$$

This is the procedure used by Coleman and Noll [22] to exploit the Clausius–Duhem inequality. It leads to the definitions of entropy and stress in terms of free energy, and to the free energy independence of the temperature gradient. The rest of the dissipation inequality

(23) $$-\frac{q}{\theta }\cdot \nabla \theta \geq 0,$$

again, takes Fourier’s law (13).

For isotropic linear thermoelasticity, the free energy per unit volume is the quadratic function

(24) $$W=\mu \varepsilon^{2}+\frac{\lambda }{2}{\left(\mathrm{tr}\varepsilon \right)}^{2}+\beta \left(\theta -\theta_{0}\right)\mathrm{tr}\varepsilon -\frac{\rho c_{p}}{2\theta_{0}}{\left(\theta -\theta_{0}\right)}^{2},$$

with Lamé coefficients λ and μ and the stress-temperature modulus β. For the stress, we have the relationship

(25) $$\sigma =\frac{\partial W}{\partial \varepsilon }=2\mu \varepsilon +\lambda \mathrm{tr}\varepsilon I+\beta \left(\theta -\theta_{0}\right)I.$$

The equation of motion in terms of displacement u is then [28]

(26) $$u_{tt}=\mu \Delta u+\left(\lambda +\mu \right)\nabla \left(\mathrm{div}u\right)+\beta \nabla \theta .$$

The balance of energy results in the heat conduction equation

(27) $$\rho c_{p}\theta_{t}-k\Delta \theta =\beta \theta_{0}\mathrm{div}u_{t}.$$

Thus, the two governing equations for linear thermoelasticity are coupled.

### 3.3. Linear Viscous Fluids

In the case of viscous fluids, we consider the isothermal situation. Here, the free energy density ψ=e−ηθ depends on the matter density ρ and the strain rate tensor D:

(28) $$\psi =\bar{\psi }\left(\rho ,D\right).$$

The Clausius–Duhem inequality in the absence of body sources has the form [28]

(29) $$-\rho \psi_{t}+\sigma :D\geq 0.$$

Applying the time derivative of the free energy density

(30) $$\psi_{t}=\frac{\partial \psi }{\partial \rho }\rho_{t}+\frac{\partial \psi }{\partial D}D_{t},$$

the decomposition of the stress tensor

(31) $$\sigma =-pI+\sigma^{v},$$

and mass conservation (1), we transform the Clausius–Duhem inequality into

(32) $$-\rho \frac{\partial \psi }{\partial D}:D_{t}-\left(-\rho^{2}\frac{\partial \psi }{\partial \rho }+p\right)\mathrm{tr}D+\sigma^{v}:D\geq 0.$$

Here, *p* is pressure and $\sigma^{v}$ is the viscous stress tensor. The satisfaction of the dissipation inequality leads to the definition of pressure

(33) $$p=\rho^{2}\frac{\partial \psi }{\partial \rho },$$

and to the free energy density independence of the strain rate tensor

(34) $$\frac{\partial \psi }{\partial D}=0.$$

The rest of the Clausius–Duhem inequality determines viscous dissipation:

(35) $$\sigma^{v}:D\geq 0.$$

For a compressible, linearly viscous (Newtonian) fluid the stress tensor is represented as [28]

(36) $$\sigma =-pI+2\mu^{*}D+\lambda^{*}\left(\mathrm{tr}D\right)I,$$

with the dilatational viscosity $\lambda^{*}$ and the shear viscosity $\mu^{*}$.

Equations of motion follow from balances of mass and momentum (in the absence of body sources) [29]:

(37) $$\begin{array}{ccc} \rho_{t} & + & \rho \;\mathrm{div}v=0, \end{array}$$

(38) $$\begin{array}{ccc} \rho v_{t} & = & -\nabla p+\mu^{*}\Delta v+\left(\lambda^{*}+\mu^{*}\right)\nabla \left(\mathrm{div}\;v\right). \end{array}$$

These equations are generally referred to as the compressible Navier–Stokes equations.

The following steps are common in the development of classical constitutive models:

- Choice of thermodynamic state space;
- Calculation of the time derivative of free energy;
- Combination of the first and second laws of thermodynamics to obtain the dissipation inequality;
- Linear solution of the dissipation inequality.

This procedure will be expanded to include the influence of internal variables.

## 4. Simple Examples of Extended Constitutive Models

The three examples above all have one feature in common: they do not “feel” the internal structure of a material. For the description of more complex situations, constitutive models have to be extended. We indicate the simplest examples of such extensions.

### 4.1. Heat Conduction in Rigid Solids

The main goal of the Fourier law extension is to eliminate infinite speed for thermal disturbance propagation. Among the numerous non-Fourier models of heat conduction [30,31], the most well-known examples are the Cattaneo–Vernotte equation:

(39) $$\tau q_{t}+q=-k\nabla \theta ,$$

and the Guyer–Krumhansl equation:

(40) $$\tau q_{t}+q=-k\nabla \theta +a\left(2\nabla \mathrm{div}q+\Delta q\right),$$

where τ denotes a relaxation time and *a* is a material parameter. Despite the use of numerous alternative models and extensive research, the question of a suitable non-Fourier heat conduction model still remains open [32,33].

### 4.2. Linear Thermoelasticity

Generalized continuum theories, such as Mindlin’s microelasticity [7] and Eringen’s polar continua [34], extend elastic constitutive models completely and can be combined with thermal models [35]. However, even for the highly reduced so-called relaxed micromorphic theory [10], the large number of additional material parameters prevents their practical implementation. This is why strain gradient theories have gained popularity [36,37]. The majority of them, however, run into causality issues [38].

The Aifantis strain gradient model [39], in particular, is distinguished by the use of the strain’s Laplacian to extend the stress–strain relationship:

(41) σ=2με+λtrε−cΔε,

where *c* is an appropriate coefficient.

This model is widely accepted in various applications [37,40,41].

### 4.3. Linear Viscous Fluids

The primary issue in hydrodynamics is turbulence [42]. There is currently no decent macroscopic description of turbulent motion [43]. Nonetheless, there are numerous rheological models that have no relationship to turbulence. A Maxwell fluid is a typical example of such a model. In this model, the stress tensor (36) is modified in the same way as in the Cattaneo–Vernotte equation [44,45]

(42) $$\tau \sigma_{t}+\sigma =-pI+2\mu^{*}D+\lambda^{*}\left(\mathrm{tr}D\right)I.$$

Regardless of their differences, the constitutive models outlined above can all be considered as results of an internal variable approach. This is shown in the subsequent sections.

## 5. Single Internal Variable Framework

We consider an internal variable that is assumed to be capable of accounting for the influence of the internal structure to extend the constitutive models. The nature of the internal variable is not specified in advance. We investigate the impact of including the internal variable in constitutive models.

The procedure outlined in Section 3 also involves the following steps:

- The derivation of evolution equations for internal variables as the solution of the dissipation inequality;
- The possible elimination of an internal variable;
- The analysis of the consequences for quadratic free energy and the linearization of results in particular cases.

### 5.1. Heat Conduction in Rigid Solids

We begin with a weakly nonlocal internal variable theory that comprises an internal variable α and its gradient into the thermodynamic state cf. [46,47]. This means that the free energy per unit volume *W* is specified as a function of the absolute temperature, the vectorial internal variable α, and its gradient [48]. With a quadratic free energy, we have

(43) $$W=-\frac{\rho c_{p}}{2\theta_{0}}{\left(\theta -\theta_{0}\right)}^{2}+\frac{1}{2}B\alpha^{2}+\frac{1}{2}C{\left(\nabla \alpha \right)}^{2},$$

where *B* and *C* are material parameters. In terms of free energy, the energy balance can be represented as

(44) $${\left(S\theta \right)}_{t}+\mathrm{div}q=-W_{t},$$

where body forces are neglected for simplicity.

Due to the dependence of the free energy on the internal variable, its time derivative is calculated using the chain rule

(45) $$\begin{array}{cc} -W_{t}= & -\frac{\partial W}{\partial \theta }\theta_{t}-\frac{\partial W}{\partial \alpha }\alpha_{t}-\frac{\partial W}{\partial \nabla \alpha }{\left(\nabla \alpha \right)}_{t}=S\theta_{t}-B\alpha :\alpha_{t}-C\nabla \alpha :{\left(\nabla \alpha_{t}\right)}^{T}. \end{array}$$

As a result, the energy balance is rewritten as follows:

(46) $${\left(S\theta \right)}_{t}+\mathrm{div}\left(q+C\nabla \alpha :\alpha_{t}\right)=S\theta_{t}+\left(C\Delta \alpha -B\alpha \right):\alpha_{t}.$$

#### 5.1.1. Dissipation Inequality

Keeping the presence of the internal variable in mind, we can write the second law of thermodynamics as

(47) $$S_{t}+\mathrm{div}\left(\frac{q}{\theta }+K\right)\geq 0,$$

where the entropy flux contains an extra entropy flux K cf. [12,48,49,50,51]. In most cases, the extra entropy flux disappears, but this is not a necessary condition.

We can rewrite the dissipation inequality by multiplying the second law (47) by temperature and taking into account energy balance Equation (46):

(48) $$\left(C\Delta \alpha -B\alpha \right):\alpha_{t}+\mathrm{div}\left(-C\nabla \alpha :\alpha_{t}+\theta K\right)-S\cdot \nabla \theta \geq 0.$$

If the extra entropy flux is chosen following the concept proposed by Maugin [12]:

(49) $$K=\frac{1}{\theta }\left(C\nabla \alpha \right):\alpha_{t},$$

then the divergence term in the dissipation inequality disappears:

(50) $$\theta \left(C\Delta \alpha -B\alpha \right):\alpha_{t}-\left(q+C\nabla \alpha :\alpha_{t}\right)\cdot \nabla \theta \geq 0.$$

The left-hand side of the obtained dissipation inequality now consists of the products of thermodynamic fluxes and forces.

#### 5.1.2. Evolution Equation for the Single Internal Variable

The standard thermodynamic approach [52] provides the solution to the dissipation inequality by representing thermodynamic fluxes $\alpha_{t}$ and $\left(q+C\nabla \alpha :\alpha_{t}\right)$ as linear functions of conjugated thermodynamic forces:

(51) $$\left(\begin{array}{c} \alpha_{t}\\ \left(q+C\nabla \alpha :\alpha_{t}\right) \end{array}\right)=M\left(\begin{array}{c} \theta \left(C\Delta \alpha -B\alpha \right)\\ -\nabla \theta \end{array}\right),$$

with

(52) $$M=\left(\begin{array}{cc} M_{11} & M_{12}\\ M_{21} & M_{22} \end{array}\right),$$

and components $M_{ij}$ of the matrix M are considered as constants for simplicity. To ensure the non-negativity of entropy production, the symmetric part of the matrix M must be positive semidefinite [53,54]:

(53) $$M_{11}\geq 0,\;M_{22}\geq 0,\;M_{11}M_{22}-\frac{{\left(M_{12}+M_{21}\right)}^{2}}{2}\geq 0.$$

As a result, the evolution equation for the internal variable α has the following form:

(54) $$\alpha_{t}=M_{11}\theta \left(C\Delta \alpha -B\alpha \right)-M_{12}\nabla \theta .$$

Accordingly, we have the generalized heat flux of the relationship:

(55) $$q+C\nabla \alpha :\alpha_{t}=M_{21}\theta \left(C\Delta \alpha -B\alpha \right)-M_{22}\nabla \theta .$$

The heat conduction equation is obtained in the framework of the single internal variable approach by replacing the heat flux in energy conservation Equation (46) with Equation (55):

(56) $$\theta S_{t}-M_{22}\Delta \theta =\left(C\Delta \alpha -B\alpha \right):\alpha_{t}-M_{21}\mathrm{div}\left[\theta (C\Delta \alpha -B\alpha )\right].$$

The right-hand side of the heat conduction equation depends on the internal variable. It is clear that the evolution equation of the internal variable (54) and heat conduction Equation (56) are coupled nonlinear parabolic equations.

#### 5.1.3. Heat Flux as Internal Variable

It is worth noting that by using heat flux as the internal variable, we obtain the following for its evolution from Equation (54):

(57) $$q_{t}=M_{11}\theta \left(C\Delta q-Bq\right)-M_{12}\nabla \theta .$$

It can be represented as an Guyer–Krumhansl-type equation:

(58) $$\frac{1}{M_{11}\theta B}q_{t}+q=-\frac{M_{12}}{M_{11}\theta B}\nabla \theta +\frac{C}{B}\Delta q.$$

When C=0, which means that the free energy is independent of the internal variable’s gradient, the evolution equation for heat flux is reduced to a Cattaneo–Vernotte-type equation:

(59) $$\frac{1}{M_{11}\theta B}q_{t}+q=-\frac{M_{12}}{M_{11}\theta B}\nabla \theta .$$

The Green–Naghdi-type equation for heat flux is reached if, on the other hand, the free energy depends only on the gradient of the internal variable and is independent of the internal variable itself (i.e., B=0):

(60) $$\frac{1}{M_{11}\theta C}q_{t}=-\frac{M_{12}}{M_{11}\theta C}\nabla \theta +\Delta q.$$

Thus, the Cattaneo–Vernotte equation and Guyer–Krumhansl equation are revealed in the framework of the single internal variable approach. The use of heat flux as an independent variable was studied in detail by Coleman et al. [55,56]. It was shown there that the Cattaneo telegrapher equation can be derived if, and only if, the internal energy does not depend on heat flux.

### 5.2. Linear Thermoelasticity

Expanding the thermoelastic state space using the tensorial internal variable α and its gradient, we can represent the free energy per unit volume *W* as a general sufficiently regular function:

(61) $$W=\bar{W}\left(\varepsilon ,\theta ,\alpha ,\nabla \alpha \right).$$

The balance of energy in terms of free energy keeps its form:

(62) $$W_{t}+{\left(\theta S\right)}_{t}-\sigma :\varepsilon_{t}+\mathrm{div}\;q=0,$$

and the second law multiplied by temperature is modified by the extra entropy flux

(63) $$\theta S_{t}+\theta \mathrm{div}\left(\frac{q}{\theta }+K\right)\geq 0.$$

The combination of Equations (62) and (63) results in the Clausius–Duhem inequality in the absence of body sources:

(64) $$-W_{t}-S\theta_{t}-\frac{q+\theta K}{\theta }\cdot \nabla \theta +\sigma :\varepsilon_{t}+\mathrm{div}\left(\theta K\right)\geq 0.$$

As previously stated, the time derivative of the free energy

(65) $$\begin{array}{c} W_{t}=\frac{\partial W}{\partial \varepsilon }:\varepsilon_{t}+\frac{\partial W}{\partial \theta }\theta_{t}+\frac{\partial W}{\partial \alpha }:\alpha_{t}+\frac{\partial W}{\partial \nabla \alpha }:{\left(\nabla \alpha \right)}_{t}, \end{array}$$

should be substituted into Equation (64), yielding

(66) $$\begin{array}{cc} \; & \left(-\frac{\partial W}{\partial \varepsilon }+\sigma \right):\varepsilon_{t}+\left(-S-\frac{\partial W}{\partial \theta }\right)\theta_{t}-\frac{\partial W}{\partial \alpha }:\alpha_{t}-\\ \; & -\frac{\partial W}{\partial \nabla \alpha }:{\left(\nabla \alpha \right)}_{t}-\frac{q+\theta K}{\theta }\cdot \nabla \theta +\mathrm{div}\left(\theta K\right)\geq 0. \end{array}$$

Again, the arbitrariness of the time rates for strain and temperature defines the stress and entropy:

(67) $$\begin{array}{c} \sigma =\frac{\partial W}{\partial \varepsilon },\;S=-\frac{\partial W}{\partial \theta }. \end{array}$$

The rest of the dissipation inequality

(68) $$-\frac{\partial W}{\partial \alpha }:\alpha_{t}-\frac{\partial W}{\partial \nabla \alpha }:{\left(\nabla \alpha \right)}_{t}-\frac{q+\theta K}{\theta }\cdot \nabla \theta +\mathrm{div}\left(\theta K\right)\geq 0,$$

can be rearranged to

(69) $$-\left(\frac{\partial W}{\partial \alpha }-\nabla \cdot \frac{\partial W}{\partial \nabla \alpha }\right):\alpha_{t}-\mathrm{div}\left(\frac{\partial W}{\partial \nabla \alpha }:\alpha_{t}+\theta K\right)-\left(\frac{q+\theta K}{\theta }\right)\cdot \nabla \theta \geq 0.$$

The extra entropy flux is selected to eliminate the divergence term, following [12]:

(70) $$K=-\frac{1}{\theta }\frac{\partial W}{\partial \nabla \alpha }:\alpha_{t}.$$

Then, the left-hand side of the dissipation inequality is transformed into the sum of the products of thermodynamic fluxes and forces:

(71) $$-\theta \left(\frac{\partial W}{\partial \alpha }-\mathrm{div}\frac{\partial W}{\partial \nabla \alpha }\right):\alpha_{t}-\left(q+\theta K\right)\cdot \nabla \theta \geq 0.$$

#### 5.2.1. Evolution Equation for Internal Variable

As before, the solution of the dissipation inequality is achieved by representing thermodynamic fluxes $\alpha_{t}$ and $\left(q+\theta K\right)$ as linear functions of conjugated thermodynamic forces. However, due to the difference in the tensorial order, both terms of Equation (71) should be non-negative separately. Therefore, the evolution equation for the internal variable α is

(72) $$\alpha_{t}=-M_{1}\theta \left(\frac{\partial W}{\partial \alpha }-\mathrm{div}\frac{\partial W}{\partial \nabla \alpha }\right).$$

For the heat flux, we have, correspondingly,

(73) $$q+\theta K=-M_{2}\nabla \theta .$$

Coefficients $M_{i}$ are considered as constants for simplicity.

#### 5.2.2. Quadratic Free Energy: Isothermal Case

In the isothermal case, the free energy per unit volume is expressed as a simplified quadratic expression due to the complexity of including the third order tensor ∇α in full:

(74) $$W=\mu \varepsilon^{2}+\frac{\lambda }{2}{\left(\mathrm{tr}\varepsilon \right)}^{2}+A\varepsilon :\alpha +\frac{1}{2}B\alpha^{2}+\frac{1}{2}C{\left(\nabla \alpha \right)}^{2}.$$

Accordingly, the derivatives of the free energy with respect to the internal variable and its gradient are

(75) $$\frac{\partial W}{\partial \alpha }=A\varepsilon +B\alpha ,\;\frac{\partial W}{\partial \nabla \alpha }=C\nabla \alpha .$$

In this case, the evolution equation for the internal variable is a reaction–diffusion-like equation:

(76) $$\alpha_{t}=-M_{1}\theta_{0}\left(A\varepsilon +B\alpha -C\Delta \alpha \right),$$

which can be found under different names in numerous applications. This type of evolution equation is commonly used in phase field theory, e.g., [57]. In the nondissipative case, we have [58]

(77) CΔα−Aε−Bα=0.

By definition, the stress tensor is the derivative of the free energy with respect to strain

(78) σ=2με+λ(trε)I+Aα,

which determines the equation of motion as

(79) $$\rho v_{t}=\mathrm{div}\sigma =\mathrm{div}\left(2\mu \varepsilon +\lambda \left(\mathrm{tr}\varepsilon \right)I\right)+A\mathrm{div}\alpha .$$

The equation of motion in terms of the displacement u is then

(80) $$u_{tt}=\mu \Delta u+\left(\lambda +\mu \right)\nabla \left(\mathrm{div}u\right)+A\mathrm{div}\alpha .$$

It is possible to eliminate the internal variable from the equation of motion. First, we express divα in terms of the displacement

(81) $$A\mathrm{div}\alpha =u_{tt}-\mu \Delta u-\left(\lambda +\mu \right)\nabla \left(\mathrm{div}u\right).$$

Accounting for Equation (77), we obtain

(82) Bdivα=CdivΔα)−Adivε.

It follows from Equation (81) that

(83) $$A\mathrm{div}\left(\Delta \alpha \right)=\Delta \left[u_{tt}-\mu \Delta u-\left(\lambda +\mu \right)\nabla \left(\mathrm{div}u\right)\right].$$

Therefore, relationship (82) is rearranged to

(84) $$B\mathrm{div}\alpha =\frac{C}{A}\Delta \left[u_{tt}-\mu \Delta u-\left(\lambda +\mu \right)\nabla \left(\mathrm{div}u\right)\right]-A\mathrm{div}\varepsilon .$$

Finally, combining the latter equations with (81), we get

(85) $$\begin{array}{cc} \; & u_{tt}-\mu \Delta u-\left(\lambda +\mu \right)\nabla \left(\mathrm{div}u\right)=\\ \; & =\frac{C}{B}\Delta \left[u_{tt}-\mu \Delta u-\left(\lambda +\mu \right)\nabla \left(\mathrm{div}u\right)\right]-\frac{A^{2}}{2B}\left(\Delta u+\nabla \mathrm{div}u\right). \end{array}$$

It is this equation that has been analyzed in studies of dispersive wave propagation in microstructured solids [59,60,61,62]. It should be noted that dispersive wave Equation (85) confronts the causality problem [38].

### 5.3. Viscous Fluids

The Navier–Stokes equations describe the motion of homogeneous isotropic fluids. We use the internal variable formalism to take into account more complicated cases. In the single internal variable theory, the free energy density depends on the density ρ, temperature θ, and the internal variable α:

(86) ψ=ψ(ρ,θ,α).

The internal variable is not predetermined in advance. However, using the dissipation inequality (29), it is possible to derive an evolution equation of the internal variable in a general form.

The material time derivative of the free energy density is calculated using the chain rule

(87) $$\psi_{t}=\frac{\partial \psi }{\partial \rho }\rho_{t}+\frac{\partial \psi }{\partial \theta }\theta_{t}+\frac{\partial \psi }{\partial \alpha }\alpha_{t}.$$

Clausius–Duhem inequality (29) is represented as follows:

(88) $$-\rho \frac{\partial \psi }{\partial \rho }\rho_{t}-\frac{\partial \psi }{\partial \alpha }:\alpha_{t}-\frac{q}{\theta }\cdot \nabla \theta +\sigma :D\geq 0.$$

The material time derivative of the density

(89) $$\rho_{t}=-\rho I:D,$$

together with pressure relation (33), transforms dissipation inequality (88) into the linear combination of products of thermodynamic fluxes and forces:

(90) $$\left(pI+\sigma \right):D-\frac{\partial \psi }{\partial \alpha }:\alpha_{t}-\frac{q}{\theta }\cdot \nabla \theta \geq 0.$$

The evolution equation for the internal variable is derived using the obtained dissipation inequality.

#### 5.3.1. Isothermal Case

We focus on the isothermal example to keep things as simple as possible. Dissipation inequality (90) in the isothermal case is reduced to

(91) $$\left(pI+\sigma \right):D-\frac{\partial \psi }{\partial \alpha }:\alpha_{t}\geq 0.$$

As before, the linear solution of dissipation inequality (91) is given by

(92) $$\left(\begin{array}{c} pI+\sigma \\ \alpha_{t} \end{array}\right)=M\left(\begin{array}{c} D\\ -\frac{\partial \psi }{\partial \alpha } \end{array}\right),$$

i.e.,

(93) $$pI+\sigma =M_{11}D+M_{12}\left(-\frac{\partial \psi }{\partial \alpha }\right),$$

(94) $$\alpha_{t}=M_{21}D+M_{22}\left(-\frac{\partial \psi }{\partial \alpha }\right).$$

where components $M_{ij}$ of the matrix M obey the same conditions of positive semidefiniteness as before. Relationships (93) and (94) establish the evolution equation for the internal variable and the thermodynamically consistent constitutive relation, respectively. These relations are not predetermined a priori or phenomenologically, but are rather the consequence of the dissipation inequality in the framework of the single internal variable theory.

In the case of a quadratic dependence of the free energy density

(95) $$\psi \left(\ldots ,\alpha \right)=\ldots +\frac{B}{2}\alpha^{2},$$

the contribution of the internal variable on the right-hand side of Equations (93) and (94) is

(96) $$-\frac{\partial \psi }{\partial \alpha }=-B\alpha .$$

#### 5.3.2. Elimination of Internal Variable

There is no need to explicitly specify the internal variable because it can be eliminated from the dissipation inequality solution. After rearranging Equation (94) with (93), we have

(97) $$\alpha_{t}=M_{21}D+\frac{M_{22}}{M_{12}}\left(pI+\sigma -M_{11}D\right).$$

The material time derivative of relation (93) yields

(98) $$p_{t}I+\sigma_{t}=M_{11}D_{t}+M_{12}\left(-B\alpha_{t}\right).$$

By substituting the time derivative of internal variable (97) into relationship (98), after some algebra, we obtain

(99) $$\begin{array}{cc} \; & \left(pI+\sigma -\frac{\det M}{M_{22}}D\right)+\frac{1}{M_{22}B}\left(p_{t}I+\sigma_{t}-M_{11}D_{t}\right)=0. \end{array}$$

The influence of the internal variable is manifested in the structure of the relationship (99) and in the value of coefficient *B* in the quadratic dependency of the free energy density (95).

We notice that when we represent each tensor as the sum of hydrostatic and deviatoric components, their contributions to the dissipation inequality are orthogonal since $A^{h}:B^{d}=0$ for arbitrary symmetric tensors. As a result, the solution of dissipation inequality (91) should be taken separately for the hydrostatic component (with the upper index *h*)

(100) $$pI+\sigma^{h}=M_{11}^{h}D^{s}+M_{12}^{h}A^{h},$$

(101) $${\alpha^{h}}_{t}=M_{21}^{h}D^{h}+M_{22}^{h}A^{h},$$

and for the deviatoric component (marked by the upper index *d*)

(102) $$\sigma^{d}=M_{11}^{d}D^{d}+M_{12}^{d}A^{d},$$

(103) $${\alpha^{d}}_{t}=M_{21}^{d}D^{d}+M_{22}^{d}A^{d}.$$

In the hydrostatic case, removing the internal variable from the solution of the dissipation inequality yields

(104) $$pI+\sigma^{h}+\frac{1}{BM_{22}^{h}}\left(p_{t}I+\sigma_{t}^{h}\right)=\frac{M_{11}^{h}}{BM_{22}^{h}}D_{t}^{h}+\frac{\det M^{h}}{M_{22}^{h}}D^{h}.$$

Similarly, the removal of the internal variable in the deviatoric example results in

(105) $$\sigma^{d}+\frac{1}{BM_{22}^{d}}\sigma_{t}^{d}=\frac{M_{11}^{d}}{BM_{22}^{d}}D_{t}^{d}+\frac{\det M^{d}}{M_{22}^{d}}D^{d}.$$

It is simple to observe that relationships (104) and (105) are simplified in the limit case B→∞

(106) $$pI+\sigma^{h}=\frac{\det M^{h}}{M_{22}^{h}}D^{h},$$

and

(107) $$\sigma^{d}=\frac{\det M^{d}}{M_{22}^{d}}D^{d}.$$

The combination of the last two equations:

(108) $$pI+\sigma =\left(\frac{\det M^{h}}{M_{22}^{h}}-\frac{\det M^{d}}{M_{22}^{d}}\right)D^{h}+\frac{\det M^{d}}{M_{22}^{d}}D,$$

can be represented as constitutive Equation (36) for the Cauchy stress

(109) $$\sigma =-pI+2\mu^{*}D+\lambda^{*}\left(\mathrm{tr}D\right)I,$$

after identifying the values of coefficients

$$\frac{\det M^{d}}{M_{22}^{d}}=2\mu^{*},\;\left(\frac{\det M^{h}}{M_{22}^{h}}-\frac{\det M^{d}}{M_{22}^{d}}\right)=3\lambda^{*},$$

since $3D^{h}=\left(\mathrm{tr}D\right)I$ by definition. The Navier–Stokes equations clearly correspond to the considered limiting case.

#### 5.3.3. Maxwell Fluids

It is now possible to observe how the Navier–Stokes equations can be expanded with the inclusion of the internal variable. To make matters easier, we set

$$M_{22}^{d}=M_{22}^{h}=M_{22},\;\tau =\frac{1}{BM_{22}}.$$

Aggregating Equations (104) and (105), we have

(110) $$\begin{array}{cc} \; & pI+\sigma =-\frac{\det M^{h}}{M_{22}}D^{h}-\frac{\det M^{d}}{M_{22}}D^{d}-\tau \left(p_{t}I+\sigma_{t}-M_{11}^{h}D_{t}^{h}-M_{11}^{d}D_{t}^{d}\right). \end{array}$$

We can represent this relationship as

(111) $$\begin{array}{cc} \; & pI+\sigma -2\mu^{*}D-\lambda^{*}\mathrm{tr}\left(D\right)I=-\tau \left(p_{t}I+\sigma_{t}-2\mu_{1}^{*}D_{t}-\lambda_{1}^{*}\mathrm{tr}{\left(D\right)}_{t}I\right), \end{array}$$

using the notation $M_{11}^{d}=2\mu_{1}^{*},\;M_{11}^{h}-M_{11}^{d}=\lambda_{1}^{*}$. Rearranging Equation (111) for the stress

(112) $$\begin{array}{cc} \; & \sigma +\tau \sigma_{t}=-pI+2\mu^{*}D+\lambda^{*}\mathrm{tr}\left(D\right)I-\tau \left(p_{t}I-2\mu_{1}^{*}D_{t}-\lambda_{1}^{*}\mathrm{tr}{\left(D\right)}_{t}I\right), \end{array}$$

we arrive at a generalization of the constitutive equation for Maxwell fluids. The comparison with early models [44,45] and with recent research [63,64,65,66,67] shows that Equation (112) has a more complex structure. Any invariant material time derivative can be employed in Equation (112) [68].

#### 5.3.4. Equations of Motion

The computation of the divergence of the stress tensor is required for the linear momentum balance

(113) $$\rho v_{t}=\mathrm{div}\;\sigma .$$

Applying divergence operator to Equation (112)

(114) $$\begin{array}{cc} \; & \mathrm{div}\;\sigma +\tau \mathrm{div}\;\sigma_{t}=-\nabla p+\mu \Delta v+\left(\lambda^{*}+\mu^{*}\right)\nabla \left(\mathrm{div}\;v\right)-\\ \; & -\tau \mathrm{div}\left(p_{t}I-2\mu_{1}^{*}D_{t}-\lambda_{1}^{*}\mathrm{tr}{\left(D\right)}_{t}I\right), \end{array}$$

we should take into account that the material time derivative and the spatial derivative do not commute, i.e.,

(115) $$\begin{array}{cc} \mathrm{div}\;\sigma_{t}= & \mathrm{div}\;\left(\frac{\partial \sigma }{\partial t}+v\cdot \left(\nabla \sigma \right)\right)=\frac{\partial \mathrm{div}\sigma }{\partial t}+\nabla v:\nabla \sigma +v\cdot \nabla \mathrm{div}\sigma =\\ \; & ={\left(\mathrm{div}\sigma \right)}_{t}+\nabla v:\nabla \sigma . \end{array}$$

Furthermore,

(116) $$\begin{array}{cc} \; & \mathrm{div}\;D_{t}={\left(\mathrm{div}D\right)}_{t}+\nabla v:\nabla D,\;\mathrm{div}\left(p_{t}I\right)={\left(\nabla p\right)}_{t}+\nabla v\cdot \nabla p,\\ \; & \mathrm{div}\left(\mathrm{tr}{\left(D\right)}_{t}I\right)={\left(\nabla \mathrm{tr}\left(D\right)\right)}_{t}+\nabla v\cdot \nabla \mathrm{tr}\left(D\right). \end{array}$$

Inserting the expressions of divergences into Equation (114), we obtain the equation

(117) $$\begin{array}{cc} \; & \rho v_{t}+\tau \left(\rho v_{tt}+\nabla v:\nabla \sigma \right)=-\nabla p+\mu^{*}\Delta v+\left(\lambda *+\mu *\right)\nabla \left(\mathrm{div}\;v\right)-\\ \; & -\tau \left(\nabla p_{t}-\mu_{1}^{*}{\left(\Delta v\right)}_{t}-\left(\lambda_{1}^{*}+\mu_{1}^{*}\right){\left(\nabla \mathrm{div}\;v\right)}_{t}+\nabla v\cdot \nabla p-\nabla v:\nabla D-\nabla v\cdot \nabla \mathrm{tr}(D\right), \end{array}$$

which can be compared with a hyperbolic perturbation of the Navier–Stokes equations (c.f. [69]). As one can see, nonlinear terms appear due to the noncommutativity of the material time derivative and the divergence operators.

#### 5.3.5. Small Relaxation Time

For small values of τ, the stress can be represented in the form of an asymptotic expansion

(118) $$\sigma =\sigma_{0}+\tau \sigma_{1}+\tau^{2}\sigma_{2}+\ldots \;.$$

The classical constitutive relation holds in zero approximation

(119) $$\sigma_{0}=-pI+2\mu^{*}D+\lambda^{*}\left(\mathrm{tr}D\right)I.$$

In the first approximation, we have

(120) $$\sigma_{1}=2\left(\mu_{1}^{*}-\mu^{*}\right)D_{t}+\left(\lambda_{1}^{*}-\lambda^{*}\right){\left(\mathrm{tr}D\right)}_{t}I.$$

The corresponding divergences are as follows:

(121) $$\mathrm{div}\;\sigma_{0}=-\nabla p+\mu^{*}\Delta v+\left(\lambda^{*}+\mu^{*}\right)\nabla \left(\mathrm{div}\;v\right),$$

(122) $$\begin{array}{cc} \; & \mathrm{div}\;\sigma_{1}=\left(\mu_{1}^{*}-\mu^{*}\right)({\left(\Delta v\right)}_{t}+\\ \; & +\nabla v:\nabla D)+\left(\lambda_{1}^{*}-\lambda^{*}+\mu_{1}^{*}-\mu^{*}\right)\left({\left(\nabla \mathrm{div}\;v\right)}_{t}+\nabla v\cdot \nabla \mathrm{tr}\left(D\right)\right). \end{array}$$

#### 5.3.6. Incompressible Fluid

Consider the case of incompressible fluids as an example. The requirement of incompressibility

(123) divv=0,

reduces the relationships for divergences to

(124) $$\mathrm{div}\;\sigma_{0}=-\nabla p+\mu^{*}\Delta v.$$

Applying the identity

(125) $${\left(\Delta v\right)}_{t}=\Delta {\left(v\right)}_{t}-\Delta \left(v\cdot \nabla v\right)+v\cdot \nabla \Delta v,$$

we can represent the divergence of the first approximation of the stress tensor as

(126) $$\mathrm{div}\;\sigma_{1}=\left(\mu_{1}^{*}-\mu^{*}\right)\left[\Delta \left(\frac{1}{\rho }\left(-\nabla p+\mu^{*}\Delta v\right)\right)-\Delta \left(v\cdot \nabla v\right)+v\cdot \nabla \Delta v+\nabla v:\nabla D\right].$$

Appeared linearized equation of motion

(127) $$\begin{array}{cc} \; & \rho v_{t}=-\nabla p+\mu^{*}\Delta v+\tau \left(\mu_{1}^{*}-\mu^{*}\right)\Delta \left(\frac{1}{\rho }\left(-\nabla p+\mu^{*}\Delta v\right)\right), \end{array}$$

can be compared to the gradient model by Aifantis [37]

(128) $$\rho v_{t}=-\nabla p+\mu^{*}\left(\Delta v-l^{2}\Delta^{2}v\right),$$

and the hyperstress model [70]

(129) $$\rho v_{t}=-\nabla p+\mu^{*}\Delta v-\zeta \Delta \Delta v.$$

Here, *l* is an internal length scale, and ζ is the difference between two constitutive moduli introduced in addition to classical viscosity $\mu^{*}$. Once more, a more complex equation of motion is produced by using internal variables.

Because they result from the solution of the dissipation inequality, the obtained extended models of fluid motion are thermodynamically consistent. Generally, the internal variable is introduced to take into account the influence of motions at a microscale that cannot be accounted for explicitly.

## 6. Dual Internal Variables

As is demonstrated, the single internal variable framework validates the thermodynamic consistency of known constitutive models for heat conduction, linear thermoelasticity, and viscous fluids. Internal variables can sometimes be removed from equations of motion. The obtained constitutive models are constrained by the parabolicity of evolution equations of internal variables. To take things a step further, the dual internal variable theory should be applied.

### 6.1. Heat Conduction in Rigid Solids

The dual internal variable theory generalizes the single internal variable theory [21,62,71]. This is specified in [72,73] for heat conduction in solids. The free energy per unit volume is assumed to be dependent on temperature, internal variables α,β, and their space gradients in this approach.

#### 6.1.1. Quadratic Free Energy

A quadratic function of its arguments is used to specify the free energy per unit volume:

(130) $$W=-\frac{\rho c_{p}}{2\theta_{0}}{\left(\theta -\theta_{0}\right)}^{2}+\frac{1}{2}B\alpha^{2}+\frac{1}{2}C{\left(\nabla \alpha \right)}^{2}+\frac{1}{2}D\beta^{2}+\frac{1}{2}F{\left(\nabla \beta \right)}^{2}.$$

Then, the time derivative of the free energy is calculated as

(131) $$\begin{array}{cc} -W_{t} & =-\frac{\partial W}{\partial \theta }\theta_{t}-\frac{\partial W}{\partial \alpha }\alpha_{t}-\frac{\partial W}{\partial \nabla \alpha }{\left(\nabla \alpha \right)}_{t}-\frac{\partial W}{\partial \beta }\beta_{t}-\frac{\partial W}{\partial \nabla \beta }{\left(\nabla \beta \right)}_{t}=\\ \; & =\;S\theta_{t}-B\alpha :\alpha_{t}-C\nabla \alpha :{\left(\nabla \alpha_{t}\right)}^{T}-D\beta :\beta_{t}-F\nabla \beta :{\left(\nabla \beta_{t}\right)}^{T}. \end{array}$$

The obtained expression for the time rate of the free energy is then used to rewrite the equation for the energy balance (Equation (44))

(132) $$\begin{array}{cc} {\left(S\theta \right)}_{t}+\mathrm{div}q & =ST_{t}-B\alpha :\alpha_{t}-C\nabla \alpha :{\left(\nabla \alpha_{t}\right)}^{T}-D\beta :\beta_{t}-F\nabla \beta :{\left(\nabla \beta_{t}\right)}^{T}. \end{array}$$

The latter expression can be rearranged to

(133) $$\begin{array}{cc} \; & {\left(S\theta \right)}_{t}+\mathrm{div}\left(q+C\nabla \alpha :\alpha_{t}+F\nabla \beta :\beta_{t}\right)=\\ \; & =S\theta_{t}+\left(C\Delta \alpha -B\alpha \right):\alpha_{t}+\left(F\Delta \beta -D\beta \right):\beta_{t}. \end{array}$$

The dissipation inequality in this case takes the form

(134) $$\begin{array}{cc} \; & \left(C\Delta \alpha -B\alpha \right):\alpha_{t}+\left(F\Delta \beta -D\beta \right):\beta_{t}+\mathrm{div}\left(C\nabla \alpha :\alpha_{t}+F\nabla \beta :\beta_{t}+\theta K\right)-\\ \; & -\frac{1}{\theta }\left(q-C\nabla \alpha :\alpha_{t}-F\nabla \beta :\beta_{t}\right)\cdot \nabla \theta \geq 0. \end{array}$$

The extra entropy flux is chosen in order to eliminate the divergence term in the dissipation inequality [12]:

(135) $$K=-\frac{1}{\theta }\left(C\nabla \alpha :\alpha_{t}+F\nabla \beta :\beta_{t}\right).$$

Due to this choice, the dissipation inequality reduces to the sum of products of thermodynamic fluxes and forces:

(136) $$\begin{array}{cc} \; & \left(C\Delta \alpha -B\alpha \right):\alpha_{t}+\left(F\Delta \beta -D\beta \right):\beta_{t}-\frac{1}{\theta }\left(q-C\nabla \alpha :\alpha_{t}-F\nabla \beta :\beta_{t}\right)\cdot \nabla \theta \geq 0. \end{array}$$

#### 6.1.2. Evolution Equations for Internal Variables

A linear solution of the dissipation inequality is constructed similarly to the case of single internal variable:

(137) $$\left(\begin{array}{c} \alpha_{t}\\ \beta_{t}\\ \left(q-C\nabla \alpha :\alpha_{t}-F\nabla \beta :\beta_{t}\right) \end{array}\right)=L\left(\begin{array}{c} \theta (C\Delta \alpha -B\alpha )\\ \theta (F\Delta \beta -D\beta )\\ -\nabla \theta \end{array}\right),$$

where matrix L

(138) $$L=\left(\begin{array}{ccc} L_{11} & L_{12} & L_{13}\\ L_{21} & L_{22} & L_{23}\\ L_{31} & L_{32} & L_{33} \end{array}\right),$$

is assumed to be positive semidefinite as mentioned above.

The evolution equations for internal variables α and β are represented as

(139) $$\begin{array}{c} \alpha_{t}=L_{11}\theta \left(C\Delta \alpha -B\alpha \right)+L_{12}\theta \left(F\Delta \beta -D\beta \right)-L_{13}\nabla \theta , \end{array}$$

(140) $$\begin{array}{c} \beta_{t}=L_{21}\theta \left(C\Delta \alpha -B\alpha \right)+L_{22}\theta \left(F\Delta \beta -D\beta \right)-L_{23}\nabla \theta , \end{array}$$

and the modified heat flux

(141) $$\begin{array}{cc} q- & C\nabla \alpha :\alpha_{t}-F\nabla \beta :\beta_{t}=L_{31}\theta \left(C\Delta \alpha -B\alpha \right)+L_{32}\theta \left(F\Delta \beta -D\beta \right)-L_{33}\nabla \theta . \end{array}$$

The heat conduction equation reads

(142) $$\begin{array}{cc} {\left(S\theta \right)}_{t} & +\mathrm{div}(L_{31}\theta \left(C\Delta \alpha -B\alpha \right)+L_{32}\theta \left(F\Delta \beta -D\beta \right)-L_{33}\nabla \theta )=\\ \; & =S\theta_{t}+\left(C\Delta \alpha -B\alpha \right):\alpha_{t}+\left(F\Delta \beta -D\beta \right):\beta_{t}. \end{array}$$

Nonlinear Equations (139)–(142) make up the closed system of equations to calculate the evolution of internal variables and temperature. In linear approximation, one of the internal variables (for example, β) can be eliminated.

#### 6.1.3. Elimination of One Internal Variable

To obtain a single equation for the internal variable α, we compute the time derivative of evolution Equation (139) ignoring the nonlinear contributions for small temperature deviations from its reference value $\theta_{0}$:

(143) $$\begin{array}{cc} \; & \alpha_{tt}=L_{11}\left(C\Delta \alpha_{t}-B\alpha_{t}\right)+L_{12}\left(F\Delta \beta_{t}-D\beta_{t}\right)-L_{13}\frac{1}{\theta_{0}}\nabla \theta_{t}. \end{array}$$

Next, the time rate of β can be expressed in terms of α by means of Equations (139) and (140):

(144) $$\beta_{t}=\frac{{\hat{L}}_{21}}{L_{12}}\left(C\Delta \alpha -B\alpha \right)+\frac{L_{22}}{L_{12}}\alpha_{t}+\frac{{\hat{L}}_{23}}{L_{12}}\frac{1}{\theta_{0}}\nabla \theta ,$$

and the time rate of the Laplacian β:

(145) $$\begin{array}{cc} \; & \Delta \beta_{t}=\frac{{\hat{L}}_{21}}{L_{12}}\Delta \left(C\Delta \alpha -B\alpha \right)+\frac{L_{22}}{L_{12}}\Delta \alpha_{t}+\frac{{\hat{L}}_{23}}{L_{12}}\frac{1}{\theta_{0}}\nabla \left(\Delta \theta \right), \end{array}$$

where the following notation is used: ${\hat{L}}_{21}=L_{21}L_{12}-L_{11}L_{22}$, ${\hat{L}}_{23}=L_{13}L_{22}-L_{23}L_{12}$.

By substituting the relationships for the time derivatives of β and its Laplacian in the expression for the second time derivative of α, the single evolution equation for the internal variable α is obtained:

(146) $$\begin{array}{cc} \alpha_{tt} & ={\hat{L}}_{21}BD\alpha -{\hat{L}}_{21}\left(CD+BF\right)\Delta \alpha -\left(L_{22}D+L_{11}B\right)\alpha_{t}+\\ \; & +\left(L_{11}C+L_{22}F\right)\Delta \alpha_{t}-{\hat{L}}_{23}D\frac{1}{\theta_{0}}\nabla \theta -L_{13}\frac{1}{\theta_{0}}\nabla \theta_{t}+\\ \; & +CF{\hat{L}}_{21}\Delta \left(\Delta \alpha \right)+F{\hat{L}}_{23}\frac{1}{\theta_{0}}\nabla \left(\Delta \theta \right). \end{array}$$

The derived evolution equation is needlessly complicated. We consider its simplified version with vanishing values of coefficients *B* and *F* in the free energy dependence to reduce the complexity of the obtained evolution equation:

(147) $$\begin{array}{cc} \alpha_{tt} & =-{\hat{L}}_{21}CD\Delta \alpha -L_{22}D\alpha_{t}+L_{11}C\Delta \alpha_{t}-{\hat{L}}_{23}D\frac{1}{\theta_{0}}\nabla \theta -L_{13}\frac{1}{\theta_{0}}\nabla \theta_{t}. \end{array}$$

Furthermore, if internal dissipation is negligible, i.e., $L_{13}=0$, $L_{11}=L_{22}=0$, we arrive at

(148) $$\begin{array}{cc} \alpha_{tt} & =-{\hat{L}}_{21}CD\Delta \alpha -{\hat{L}}_{23}D\frac{1}{\theta_{0}}\nabla \theta . \end{array}$$

It is a hyperbolic equation for the evolution of internal variable α if $\left(-{\hat{L}}_{21}CD\right)$ is positive.

In the linear approximation, the heat conduction equation can be rewritten as

(149) $$\begin{array}{c} \rho c_{p}\theta_{t}-L_{33}\Delta \theta =\mathrm{div}\left(L_{31}\theta_{0}\left(C\Delta \alpha \right)+\frac{L_{32}}{L_{12}}\alpha_{t}\right). \end{array}$$

Equations (148) and (149) are coupled. Together, they describe the heat conduction process in microstructured bodies, for example. In this case, the internal variable α represents the microtemperature, i.e., the temperature fluctuation around the macrotemperature. While the heat conduction equation for the macrotemperature remains parabolic, the evolution equation for the internal variable α is hyperbolic in contrast to the case of the single internal variable. This is the key distinction between frameworks with single and dual internal variables.

### 6.2. Linear Thermoelasticity

For linear thermoelasticity, the internal variable theory is easily generalized to the situation where there are two internal variables. Consider the free energy per unit volume *W* as a function of two internal variables, α and β, each of which is a second-order tensor:

(150) $$W=\bar{W}\left(\varepsilon ,\theta ,\alpha ,\nabla \alpha ,\beta ,\nabla \beta \right).$$

The energy balance in terms of free energy preserves its structure:

(151) $$W_{t}+{\left(\theta S\right)}_{t}-\sigma :\varepsilon_{t}+\mathrm{div}\;q=0,$$

and the second law multiplied by temperature is modified by the extra entropy flux:

(152) $$\theta S_{t}+\theta \mathrm{div}\left(\frac{q}{\theta }+K\right)\geq 0.$$

The combination of Equations (151) and (152) results in the Clausius–Duhem inequality in the absence of body sources

(153) $$-W_{t}-S\theta_{t}-\frac{q+\theta K}{\theta }\cdot \nabla \theta +\sigma :\varepsilon_{t}+\mathrm{div}\left(\theta K\right)\geq 0.$$

As previously, the time derivative of the free energy

(154) $$\begin{array}{c} W_{t}=\frac{\partial W}{\partial \varepsilon }:\varepsilon_{t}+\frac{\partial W}{\partial \theta }\theta_{t}+\frac{\partial W}{\partial \alpha }:\alpha_{t}+\frac{\partial W}{\partial \nabla \alpha }:{\left(\nabla \alpha \right)}_{t}+\frac{\partial W}{\partial \beta }:\beta_{t}+\frac{\partial W}{\partial \nabla \beta }:{\left(\nabla \beta \right)}_{t}, \end{array}$$

should be substituted into Equation (153), yielding

(155) $$\begin{array}{cc} \; & \left(-\frac{\partial W}{\partial \varepsilon }+\sigma \right):\varepsilon_{t}+\left(-S-\frac{\partial W}{\partial \theta }\right)\theta_{t}-\frac{\partial W}{\partial \alpha }:\alpha_{t}-\frac{\partial W}{\partial \nabla \alpha }:{\left(\nabla \alpha \right)}_{t}-\\ \; & -\frac{\partial W}{\partial \beta }:\beta_{t}-\frac{\partial W}{\partial \nabla \beta }:{\left(\nabla \beta \right)}_{t}-\frac{q+\theta K}{\theta }\cdot \nabla \theta +\mathrm{div}\left(\theta K\right)\geq 0. \end{array}$$

Again, the arbitrariness of the time rates for strain and temperature defines the stress and entropy:

(156) $$\begin{array}{c} \sigma =\frac{\partial W}{\partial \varepsilon },\;S=-\frac{\partial W}{\partial \theta }. \end{array}$$

The rest of the dissipation inequality

(157) $$-\frac{\partial W}{\partial \alpha }:\alpha_{t}-\frac{\partial W}{\partial \nabla \alpha }:{\left(\nabla \alpha \right)}_{t}-\frac{\partial W}{\partial \beta }:\beta_{t}-\frac{\partial W}{\partial \nabla \beta }:{\left(\nabla \beta \right)}_{t}-\frac{q+\theta K}{\theta }\cdot \nabla \theta +\mathrm{div}\left(\theta K\right)\geq 0,$$

can be rearranged to

(158) $$\begin{array}{cc} \; & -\left(\frac{\partial W}{\partial \alpha }-\mathrm{div}\frac{\partial W}{\partial \nabla \alpha }\right):\alpha_{t}-\left(\frac{\partial W}{\partial \beta }-\mathrm{div}\frac{\partial W}{\partial \nabla \beta }\right):\beta_{t}-\\ \; & -\mathrm{div}\left(\frac{\partial W}{\partial \nabla \alpha }:\alpha_{t}+\frac{\partial W}{\partial \nabla \beta }:\beta_{t}+\theta K\right)-\left(\frac{q+\theta K}{\theta }\right)\cdot \nabla \theta \geq 0. \end{array}$$

The extra entropy flux is selected to eliminate the divergence term, following [12]:

(159) $$K=-\frac{1}{\theta }\frac{\partial W}{\partial \nabla \alpha }:\alpha_{t}-\frac{1}{\theta }\frac{\partial W}{\partial \nabla \beta }:\beta_{t}.$$

Then, the left-hand side of the dissipation inequality is transformed to the sum of products of thermodynamic fluxes and forces:

(160) $$-\theta \left(\frac{\partial W}{\partial \alpha }-\mathrm{div}\frac{\partial W}{\partial \nabla \alpha }\right):\alpha_{t}-\theta \left(\frac{\partial W}{\partial \beta }-\mathrm{div}\frac{\partial W}{\partial \nabla \beta }\right):\beta_{t}-\left(q+\theta K\right)\cdot \nabla \theta \geq 0.$$

#### 6.2.1. Dissipation Inequality: Isothermal Case

Until now, the introduction of a dual internal variable looks like a repetition of the single internal variable formalism. However, the dissipation inequality is more general even in the isothermal case

(161) $$-\left(\frac{\partial W}{\partial \alpha }-\mathrm{div}\frac{\partial W}{\partial \nabla \alpha }\right):\alpha_{t}-\left(\frac{\partial W}{\partial \beta }-\mathrm{div}\frac{\partial W}{\partial \nabla \beta }\right):\beta_{t}\geq 0,$$

and its linear solution

(162) $$\left(\begin{array}{c} \alpha_{t}\\ \beta_{t} \end{array}\right)=M\left(\begin{array}{c} -\left(\frac{\partial W}{\partial \alpha }-\mathrm{div}\frac{\partial W}{\partial \nabla \alpha }\right)\\ -\left(\frac{\partial W}{\partial \beta }-\mathrm{div}\frac{\partial W}{\partial \nabla \beta }\right) \end{array}\right),$$

where components $M^{11},\ldots ,M^{22}$ of the linear operator M are dependent on state variables [74]. As previously stated, the matrix *M* should be positive semidefinite.

#### 6.2.2. Quadratic Free Energy

Representing the free energy per unit volume as the quadratic function

(163) $$W=\mu \varepsilon^{2}+\frac{\lambda }{2}{\left(\mathrm{tr}\varepsilon \right)}^{2}+A\varepsilon :\alpha +\frac{1}{2}B\alpha^{2}+\frac{1}{2}C{\left(\nabla \alpha \right)}^{2}+\frac{1}{2}D\beta^{2}+\frac{1}{2}F{\left(\nabla \beta \right)}^{2},$$

we obtain four derivatives of the free energy

(164) $$\frac{\partial W}{\partial \alpha }=A\varepsilon +B\alpha ,\;\frac{\partial W}{\partial \nabla \alpha }=C\nabla \alpha ,\;\frac{\partial W}{\partial \beta }=D\beta ,\;\frac{\partial W}{\partial \nabla \beta }=F\nabla \beta .$$

The evolution equations for internal variables are specified as

(165) $$\alpha_{t}=M^{11}\left(C\Delta \alpha -A\varepsilon -B\alpha \right)+M^{12}\left(F\Delta \beta -D\beta \right),$$

(166) $$\beta_{t}=M^{21}\left(C\Delta \alpha -A\varepsilon -B\alpha \right)+M^{22}\left(F\Delta \beta -D\beta \right).$$

As before, it is possible to derive a single evolution equation for the internal variable α:

(167) $$\begin{array}{cc} \; & \alpha_{tt}=M^{11}\left(C\Delta \alpha_{t}-A\varepsilon_{t}-B\alpha_{t}\right)+\\ \; & +F[M^{22}\Delta \alpha_{t}+\left(M^{12}M^{21}-M^{11}M^{22}\right)\Delta \left(C\Delta \alpha -A\varepsilon -B\alpha \right)]-\\ \; & -D[M^{22}\alpha_{t}+\left(M^{12}M^{21}-M^{11}M^{22}\right)\left(C\Delta \alpha -A\varepsilon -B\alpha \right)]. \end{array}$$

A simplified version of this evolution equation is obtained for B=0 and F=0:

(168) $$\begin{array}{cc} \; & \alpha_{tt}=M^{11}\left(C\Delta \alpha_{t}-A\varepsilon_{t}\right)-D\left[M^{22}\alpha_{t}+\left(M^{12}M^{21}-M^{11}M^{22}\right)\left(C\Delta \alpha -A\varepsilon \right)\right]. \end{array}$$

In the nondissipative case ($M^{11}=M^{22}=0$ and $M^{12}=-M^{21}$), the evolution equation is reduced to a hyperbolic equation:

(169) $$\begin{array}{cc} \; & \alpha_{tt}=-DM^{12}M^{21}\left(C\Delta \alpha -A\varepsilon \right). \end{array}$$

By definition, the stress tensor is the derivative of the free energy with respect to strain:

(170) σ=2με+λ(trε)I+Aα,

which determined the equation of motion as

(171) $$\rho v_{t}=\mathrm{div}\sigma =\mathrm{div}\left(2\mu \varepsilon +\lambda \left(\mathrm{tr}\varepsilon \right)I\right)+A\mathrm{div}\alpha .$$

The equation of motion in terms of displacement u is then

(172) $$\begin{array}{cc} \; & {\left[\rho u_{tt}-\mu \Delta u-\left(\lambda +\mu \right)\nabla \left(\mathrm{div}u\right)\right]}_{tt}=\\ \; & =-DM^{12}M^{21}\left\{C\Delta \left[\rho u_{tt}-\mu \Delta u-\left(\lambda +\mu \right)\nabla \left(\mathrm{div}u\right)\right]-\frac{A^{2}}{2}\left[\Delta u+\nabla \left(\mathrm{div}u\right)\right]\right\}. \end{array}$$

This is a hyperbolic equation of higher order as is discussed in [58,59,60,61,62,71,75,76,77]. It is obvious that Equation (172) has no issues with causality and is valid for the propagation of dispersive waves [77]. In contrast, the causality issue is unavoidable in the single internal variable approach.

### 6.3. Viscous Fluids

Now, we study the difference between single and dual internal variable descriptions in the case of viscous fluids. In the dual internal variable approach, it is supposed that the free energy density depends on internal variables α and β:

(173) ψ=ψ(ρ,θ,α,β).

We compute the time derivative of the free energy:

(174) $$\begin{array}{cc} \; & \psi_{t}=\frac{\partial \psi }{\partial \rho }\rho_{t}+\frac{\partial \psi }{\partial \theta }\theta_{t}+\frac{\partial \psi }{\partial \alpha }\alpha_{t}+\frac{\partial \psi }{\partial \beta }\beta_{t}, \end{array}$$

to use it in the Clausius–Duhem inequality:

(175) $$-\rho \psi_{t}-\rho \eta \theta_{t}-\left(\frac{q}{\theta }\right)\cdot \nabla \theta +\sigma :D\geq 0.$$

If the free energy density is quadratic

(176) $$\psi \left(\ldots ,\alpha ,\nabla \beta \right)=\ldots +\frac{B}{2}\alpha^{2}+\frac{D}{2}\beta^{2},$$

then the dissipation inequality is transformed into the sum of products of thermodynamic fluxes and forces taking into account Equation (33):

(177) $$\begin{array}{cc} \; & \left(pI+\sigma \right):D+\left(-B\alpha \right):\alpha_{t}+\left(-D\beta \right):\beta_{t}-\frac{q}{\theta }\cdot \nabla \theta \geq 0. \end{array}$$

As before, the representation of thermodynamic fluxes as linear functions of conjugated thermodynamic forces leads to the solution of the dissipation inequality (177).

#### 6.3.1. Isothermal Case

The dissipation inequality is even simpler in the isothermal situation

(178) $$\begin{array}{cc} \; & \left(pI+\sigma \right):D+\left(-B\alpha \right):\alpha_{t}+\left(-D\beta \right):\beta_{t}\geq 0. \end{array}$$

The linear solution to the dissipation inequality is obtained in the same manner as in the case of a single internal variable:

(179) $$\left(\begin{array}{c} pI+\sigma \\ \alpha_{t}\\ \beta_{t} \end{array}\right)=L\left(\begin{array}{c} D\\ \left(-B\alpha \right)\\ \left(-D\beta \right) \end{array}\right),$$

where

(180) $$L=\left(\begin{array}{ccc} L_{11} & L_{12} & L_{13}\\ L_{21} & L_{22} & L_{23}\\ L_{31} & L_{32} & L_{33} \end{array}\right),$$

with the same condition concerning the positive semidefiniteness of the matrix L as for the single internal variable case. As a result, the constitutive relationship for stress is depicted as

(181) $$\begin{array}{c} pI+\sigma =L_{11}D+L_{12}\left(-B\alpha \right)+L_{13}\left(-D\beta \right). \end{array}$$

Evolution equations for internal variables α and β take the form

(182) $$\alpha_{t}=L_{21}D+L_{22}\left(-B\alpha \right)+L_{23}\left(-D\beta \right),$$

(183) $$\beta_{t}=L_{31}D+L_{32}\left(-B\alpha \right)+L_{33}\left(-D\beta \right).$$

Both the constitutive relationship (181) and the evolution equations are coupled. The internal variables α and β are dual, as shown by Equations (182) and (183).

#### 6.3.2. Elimination of One Internal Variable

We can eliminate one of the two internal variables. We take the time differentiation of Equation (182) to obtain

(184) $$\begin{array}{cc} \; & \alpha_{tt}=L_{21}D_{t}+L_{22}\left(-B\alpha_{t}\right)+L_{23}\left(-D\beta_{t}\right), \end{array}$$

and then develop expressions for $\beta_{t}$ in terms of α using the relation

(185) $$\begin{array}{cc} \; & L_{23}\left(-D\beta \right)=\alpha_{t}-L_{21}D-L_{22}\left(-B\alpha \right), \end{array}$$

which follows from Equation (182). Thus, we have

(186) $$\begin{array}{cc} \; & \beta_{t}=L_{31}D+\frac{L_{32}L_{23}-L_{22}L_{33}}{L_{23}}\left(-B\alpha \right)+\frac{L_{33}}{L_{23}}\left(\alpha_{t}-L_{21}D\right), \end{array}$$

We obtain the evolution equation of the internal variable α in its own terms by substituting equality (186) into Equation (184):

(187) $$\begin{array}{cc} \; & \alpha_{tt}=L_{21}D_{t}+L_{22}\left(-B\alpha_{t}\right)-D\left(L_{31}L_{23}D+\left(L_{32}L_{23}-L_{22}L_{33}\right)\left(-B\alpha \right)\right)-\\ \; & -DL_{33}\left(\alpha_{t}-L_{21}D\right). \end{array}$$

The internal variable α alone can also be used to rewrite constitutive relation (181):

(188) $$\begin{array}{cc} pI+\sigma & =L_{11}D+L_{12}\left(-B\alpha \right)+\frac{L_{13}}{L_{23}}\left(\alpha_{t}-L_{21}D-L_{22}\left(-B\alpha \right)\right). \end{array}$$

The last equation can be resolved for $\alpha_{t}$:

(189) $$\begin{array}{cc} \alpha_{t}= & \frac{L_{23}}{L_{13}}\left(pI+\sigma \right)-\frac{\left(L_{11}L_{23}-L_{13}L_{21}\right)}{L_{13}}D-\frac{\left(L_{12}L_{23}-L_{13}L_{22}\right)}{L_{13}}\left(-B\alpha \right), \end{array}$$

which gives another expression for the evolution equation of the internal variable α:

(190) $$\begin{array}{cc} \alpha_{tt}= & \frac{L_{23}}{L_{13}}{\left(pI+\sigma \right)}_{t}-\frac{\left(L_{11}L_{23}-L_{13}L_{21}\right)}{L_{13}}D_{t}-\frac{\left(L_{12}L_{23}-L_{13}L_{22}\right)}{L_{13}}\left(-B\alpha_{t}\right). \end{array}$$

When we compare Equations (187) and (190), we get the solution to the dissipation inequality in the form

(191) $$\begin{array}{cc} \; & {\left(pI+\sigma \right)}_{t}-L_{11}D_{t}-L_{12}\left(-B\alpha_{t}\right)=\\ \; & -D\left(L_{33}\left(pI+\sigma \right)+\left(L_{13}L_{31}-L_{11}L_{33}\right)D+\left(L_{32}L_{13}-L_{12}L_{33}\right)\left(-B\alpha \right)\right). \end{array}$$

In the dual internal variable approach, Equations (187) and (191) provide the evolution equation for the internal variable α and the constitutive relation for fluid flow.

#### 6.3.3. Asymptotics

It is difficult to analyze the obtained constitutive relationship in its entirety. However, the relationship is divided into two parts that can be considered separately. In fact, the coefficient *D* describes the impact of the second internal variable on the overall behavior. If this coefficient is small, than the right-hand side of Equation (191) can be neglected, which results in

(192) $$\begin{array}{c} {\left(pI+\sigma \right)}_{t}-L_{11}D_{t}=L_{12}\left(-B\alpha_{t}\right). \end{array}$$

The evolution equation for the internal variable α is then reduced to

(193) $$\begin{array}{cc} \; & \alpha_{t}-L_{21}D-L_{22}\left(-B\alpha \right)=0, \end{array}$$

and the constitutive relation of Equation (188) is represented as

(194) $$\begin{array}{c} pI+\sigma =L_{11}D+L_{12}\left(-B\alpha \right). \end{array}$$

We can eliminate the internal variable α by combining the obtained relationships

(195) $$\begin{array}{c} {\left(pI+\sigma \right)}_{t}-L_{11}D_{t}=BL_{22}\left(pI+\sigma \right)+B\left(L_{12}L_{21}-L_{22}L_{11}\right)D, \end{array}$$

which produces a variant of the constitutive equation for the case of a single internal variable, as expected.

In the opposite case, when the coefficient *D* is large, we obtain a different constitutive equation:

(196) $$L_{33}\left(pI+\sigma \right)+\left(L_{13}L_{31}-L_{11}L_{33}\right)D+\left(L_{32}L_{13}-L_{12}L_{33}\right)\left(-B\alpha \right)=0.$$

The equation of motion for large values of *D* is

(197) $$\begin{array}{cc} \; & \rho v_{t}=-\nabla p+\frac{L_{13}L_{31}-L_{11}L_{33}}{2L_{33}}\Delta v-B\frac{L_{32}L_{13}-L_{12}L_{33}}{L_{33}}\mathrm{div}\;\alpha . \end{array}$$

This equation resembles the Navier–Stokes equation of motion. The last term on the right-hand side, however, is dependent on the divergence of the internal variable. The situation reduces to the classical incompressible case in the absence of internal variables.

The evolution equation for the internal variable α (187) is based on Equation (185), which can be hyperbolic if the internal variable β is interpreted as the gradient ∇α:

(198) $$\begin{array}{cc} \; & L_{23}\left(-D\nabla \alpha \right)=\alpha_{t}-L_{21}D-L_{22}\left(-B\alpha \right), \end{array}$$

and coefficients $L_{23}$ and *D* have opposite signs.

## 7. Conclusions

Constitutive modeling with internal variables is based on the premise that traditional constitutive models are merely a first approximation in describing actual phenomena and should be enhanced for broader applicability. Internal variables represent what we are unable to directly describe phenomenologically. The widely exploited phase-field approaches [78,79,80] are, in fact, examples of the use of internal variables [57,81]. The same is true for the gradient elasticity theory [58]. Moreover, the Mindlin microelasticity and Cosserat microrotation theories can be interpreted in terms of dual internal variables [71]. The duality of internal variables refers to the use of two equally valuable coupled internal variables to describe the same phenomenon rather than the use of multiple internal variables to describe different phenomena [21].

The paper presents a thermodynamically consistent framework for treating internal variables with as little a priori knowledge as possible. This framework is based on the exploitation of the Clausius–Duhem inequality. Because the considered internal variables are “observable but not controllable”, only the Onsagerian procedure with the use of the extra entropy flux is appropriate for deriving of evolution equations for internal variables.

The most noticeable effect is the clear distinction between constitutive models with single and dual internal variables. It is shown that the evolution equations for internal variables are parabolic in the case of a single internal variable and may be hyperbolic if dual internal variables are employed. This conclusion holds true for all considered examples.

In the case of heat conduction in rigid solids, the constitutive model with a single internal variable provides both the Cattaneo–Vernotte and the Guyer–Krumhansl equations if the internal variable is interpreted as heat flux. However, both the heat conduction equation and the internal variable evolution equation are parabolic. Only in the case of dual internal variables is it possible to obtain a hyperbolic evolution equation for the internal variable after one of them is eliminated. The remaining internal variable can be interpreted as a microtemperature (the fluctuation around mean temperature). Although the heat conduction equation remains parabolic, the coupling with the microtemperature evolution causes wave-like heat propagation [75,82,83].

The constitutive models for thermoelasticity are illustrated using dispersive wave propagation as an example. The internal variables here can be interpreted as microdeformations [62,71,75]. In the case of a single internal variable, the corresponding models confront the issue of causality [38]. This issue can only be eliminated by employing dual internal variables [77].

The extension of the Navier–Stokes equations yields constitutive equations for Maxwell fluids in the framework of a single internal variable [84]. In contrast, the dual internal variable approach is able to produce Reynolds-type equations of motion [43] with a possibly hyperbolic evolution equation for an internal variable.

It should be emphasized that the constitutive models with internal variables are typically nonlinear and generalize existing models [85,86,87,88]. This opens up a wide range of possibilities for applying internal variable theory to constitutive modeling of complex phenomena. The paper includes simplified examples of such models so that they can be compared to known models.

## Data Availability

Not applicable.
